# A screen for peptide agonists of the G-CSF receptor

**DOI:** 10.1186/1756-0500-4-194

**Published:** 2011-06-15

**Authors:** Nadine Conzelmann, Armin Schneider

**Affiliations:** 1 SYGNIS Bioscience, Im Neuenheimer Feld 515, 69120 Heidelberg, Germany

## Abstract

**Background:**

Granulocyte-colony stimulating factor (G-CSF) is one of the most important pharmacologically used proteins. Potential uses beyond the stimulation of neutrophilic granulocytes are the treatment of CNS disorders. Disadvantages of the G-CSF protein as a drug are its moderate plasma half-life time and considerable production costs. We therefore conducted a screen for peptide agonists derived from the sequence of human G-CSF.

**Findings:**

Despite of the high sensitivity of our screening system we could not detect any positive hits in a single peptide approach. In a multiplex approach using a permutation of any combination of 10 different peptides we could also not detect a positive block.

**Conclusions:**

We conclude that larger coherent parts of the protein or dimerising peptides may be needed to achieve activation of the receptor.

## Background

Granulocyte-colony stimulating factor (G-CSF) is one the most widely employed protein drugs. It is mostly used for counteracting neutropenia in patients receiving chemotherapy [1], but also for stem cell harvesting [2,3], and as add-on to anti-infectious therapy. G-CSF is a glycoprotein that binds in 2:2 ligand: receptor stoichiometry to its cytokine like receptor (G-CSF-R) which recruits in turn Janus kinases (JAKs), a family of protein tyrosine kinases. These kinases phosphorylate the receptor and also themselves starting by that a multiple signalling cascade, which involves amongst others STAT1 and 3, PI3K/Akt and the Ras/Mek/Erk1/2 pathway [4-8].

Recently we and others have defined a novel spectrum of G-CSF activities in the central nervous system where it acts on neurons. For example, it was shown that G-CSF can reduce the infarct size in animal stroke models [9-11] and that it has significant beneficial effects on the motor performance as well as on the overall survival in a mouse model for Amyotrophic lateral sclerosis (ALS) [12]. G-CSF is therefore clinically explored for several neurological diseases, such as stroke [13,14].

Disadvantages of the G-CSF protein for a potential lifetime continuous therapy as in the case of chronic neurodegenerative conditions are its limited plasma half-life time (~4 h), the relatively high costs, and chronic effects on the hematopoietic system. Pegylated forms of G-CSF are available that have a much extended half-life, however, it is unclear at present if those modifications hinder passage of the blood-brain-barrier (BBB).

We therefore decided to conduct a screen searching for peptides derived from the human G-CSF peptide sequence with agonistic activity. Peptides would be considerably cheaper to produce, be potentially suitable for delivery methods other than subcutaneous injection, and might also show improved neuronal selectivity.

It was previously shown for a number of protein receptors that this is a feasible concept in principle. For example, peptides derived from the NCAM (neural cell adhesion molecule) sequence, a cell surface glycoprotein that belongs to the Ig superfamily, act agonistically on the fibroblast growth factor (FGF) receptor [15-17]. NCAM is involved in the formation of neuronal connections during development and modulates synaptic plasticity associated with regeneration and learning [18-20]. Several mimetic peptides have been derived from its protein structure, like C3 by combinatorial chemistry from the NCAM Ig1 module [21], the BCL motif from the second NCAM fibronectin type III module [22] and P2, a 12 amino acid sequence localized in the FG loop of the second Ig module of NCAM [23]. All these peptides are potent mimetics of NCAM and therefore attractive compounds in the development of therapies in neurodegenerative disorders.

Moreover, agonistic peptides have been found for the EPO receptor (EPO-R), functionally very close to the G-CSF receptor (G-CSF-R), both derived from the EPO sequence [24] or fully novel [25,26]. Indeed, one of the agonistic peptides has entered clinical development [27]. This agonistic peptide for the EPO-R (EMP-1) was discovered by phage display and is a cyclic peptide of 20 residues, which has nothing in common with the original EPO protein structure. EMP-1 can compete with EPO at micromolar concentrations by binding as a dimer to the extracellular domain of the EPO-R and by activating the receptor by induced dimerization. This was also shown by the crystal structure of the complex of EMP-1 with recombinant soluble EPO-receptor. In two animal models for erythropoesis EMP-1 also exhibited activity [26,28]. In further development steps EMP-1 has been chemically modified through covalent linkage of polyethylene glycol resulting in the EMP-1 related dipeptide Hematide™ [26]. Hematide™ obtained an increased half life and has been shown to be safe in healthy volunteers by increasing haemoglobin levels for one month [29]. A clinical trial phase III has just been completed in 2010.

In 1998 the nonpeptidyl small molecule SB 247464 was identified as a ligand of the murine G-CSF receptor in a high-throughput cell based screening approach by Tian and colleagues [30]. Like G-CSF, SB 247464 induced tyrosine phosphorylation of multiple signaling proteins, and stimulated primary murine bone marrow cells by oligomerizing the receptor chains. Nonetheless the synthetic compound showed lower potency compared to G-CSF and bound exclusively to the murine but not the human G-CSF-R [30]. Up to now no other agonistic small molecules or peptides for the human G-CSF-R have been identified or described.

Thus we conducted a sensitive screen for peptide agonists derived from the sequence of human G-CSF employing the murine myeloblastic cell line NFS-60. This suspension cell line expands rapidly in culture and is dependent on G-CSF of either murine or human origin, or alternatively on M-CSF (Macrophage colony-stimulating factor) or IL-3 (Interleukin-3) [31]. Our assay read-out was viability of these cells in the absence of G-CSF.

## Methods

### Peptide Library

For the design of the peptide library the protein sequence of human G-CSF [NCBI: NP_000750] was used. The first 29 amino acids of the G-CSF protein (the signal sequence) were not considered for the library. For the remaining 178 amino acids of the G-CSF sequence we designed an overlapping peptide library for G-CSF with the help of the PepScreen^® ^Library Design Tool [32]. We chose an overlapping 20 mer peptide library with a shift of 2 amino acids per peptide, resulting in a total of 80 peptides. The peptide library was ordered in crude quality with a minimum content of 30% intact peptide that was analyzed by MS and HPLC by the provider (Genscript USA Inc., Piscataway, NJ, USA). Because of the on average high hydrophobicity of the peptides they were dissolved in DMSO at a concentration of 1 mM and stored at -80°C.

### Cell line

The murine myeloblastic cell line NFS-60 expands rapidly in culture and is dependent on G-CSF of either murine or human origin, or alternatively on M-CSF (Macrophage colony-stimulating factor) or IL-3 (Interleukin-3) [31]. The cell line was cultured as suspension culture in RPMI 1640 medium supplemented with 5.1 ml L-glutamine (200 mM), 10% fetal bovine serum, 1x P/S, 10 μM β-Mercaptoethanol and 2 ng/ml G-CSF at 37°C and with a CO_2 _fraction of 5%.

### Establishing viability curves for G-CSF dependency

NFS-60 cells were washed twice with cell culture medium containing no G-CSF and were then plated at a total density of 1 × 10^4^/well in 96-well plates. Subsequently human recombinant G-CSF (AX200, Dr. Reddy's Laboratories, Hyderabad, India; 300 μg/ml) was added to the cells at increasing concentrations, and cells were incubated for 48 h at 37°C. Viability of the cells was detected using the MultiTox-Fluor Multiplexicity Assay (Promega, Mannheim, Germany) as described by the provider. Fluorescence was measured using the plate reader FLUOstar (BMG Labtech, Offenburg, Germany) at 390 nm/520 nm.

### Screening of single peptides

96-well plates were prepared with 8 peptides with 8 replicates/plate, 2 negative controls (G-CSF buffer (10 mM Acetic Acid, 250 mM Sorbitol, 0.004% Tween 80)) at 8 replicates/plate, and two positive controls, one at the lower end of sensitivity, and one at a 100% viability level (each at 8 replicates). In order to avoid evaporation of the edging wells and to have the same conditions for all probes the samples with their eight replicates were pipetted in a diagonal pattern on a 96-well plate automatically by the MultiProbeII EX from Packard [Additional file 1].

NFS-60 cells were washed twice with cell culture medium containing no G-CSF and were then plated in a 96-well plate at a total cell number of 1 × 10^4^/well. The peptide plate was then transferred by the Liquidator^96 ^(Steinbrenner Laborsysteme GmbH) to this plate and incubated at 37°C for 48 hours. Final DMSO concentrations were 0.2% for the 1 μM screen, and 2% for the 10 μM screen. For detection of viability the MultiTox-Fluor Multiplexicity Assay from Promega was used as described by the provider. The fluorescence of the plate was measured in a fluorescence reader (FLUOstar) at 390 nm and 520 nm.

### Screening of multiple peptides

Up to 20 peptides per well were tested in a multiplex approach. The 80 peptides of the library were arranged in eight groups per ten peptides. Permutations of all combinations of two peptide groups were tested.

### Statistical Analyses

All results were evaluated in EXCEL. For analysis of the data a student's t-test against both negative controls was performed. A p-value <0.05 was considered significant.

## Results

### Development of a screening assay based on G-CSF dependency

**Figure 1:**
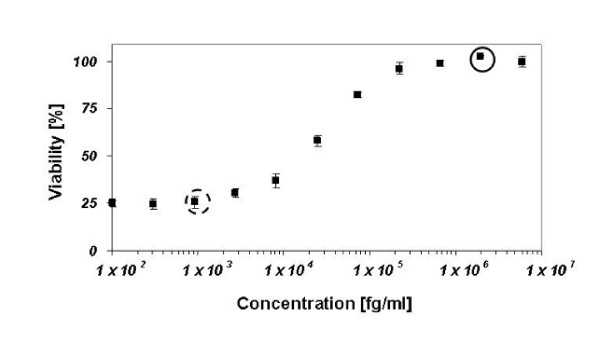
**Titration curve of G-CSF on the G-CSF dependant cell line NFS-60**. NFS-60 were incubated with increasing concentrations of G-CSF for 48 h. The resulting viability curve was used to choose positive controls for the subsequent screen (0.1 nM and 0.1 pM G-CSF). The concentration of 0.1 nM (closed circle) corresponds to 100% viability of the cells. The concentration of 0.1 pM (dashed circle) reflects the lowest significant increase in viability observed.

We used the G-CSF-dependent cell line NFS-60 for screening a library of G-CSF-derived peptides. First we established a survival curve over a wide concentration range of G-CSF (Figure 1) with a resulting EC50 value about 0.8 pM or18 pg/ml G-CSF. For the subsequent peptide screen we chose two positive controls, one at 100% viability (0.1 nM or 2 ng/ml G-CSF), Figure 1 closed circle), and one at the lowest G-CSF concentration that still had a significant effect on viability (0.1 pM or 2.7 pg/ml G-CSF, Figure 1 dashed circle). These two concentrations first guarantee a constant sensitivity of the assay, and would allow estimating the relative potency of hits.

### Screening of single peptides

**Figure 2:**
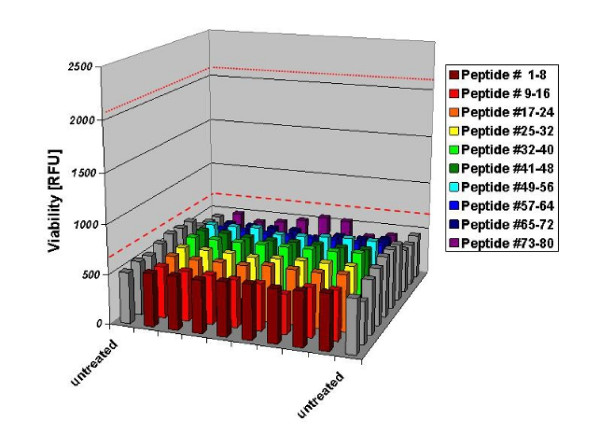
**Results of the G-CSF peptide screening at 1 μM**. Controls on each 96-well plate included two negative controls without G-CSF and two positive controls with a molarity of 0.1 nM and 0.1 pM G-CSF. The mean of the two positive controls are indicated as red lines (red dotted line = first positive control, red dashed line = second positive control). None of the 80 peptides screened showed a significant increase in viability compared to the negative controls. (n = 8 replicates each).

We designed a 20-mer peptide library derived from the human G-CSF sequence with a sliding overlap of 2 amino acids. We first conducted a screen at a concentration of 1 μM per peptide, a 10 ^4^-fold higher molarity than the lower positive control. Results for the 80 peptides are given in Figure 2 with means of the two positive controls over all screened plates indicated as red lines in the diagram (dotted, lower positive control; dashed, upper positive control). Negative controls are shown as grey bars. The mean standard deviation over 8 replicates of the negative controls was +/- 6.37%, of the screened peptides +/- 5.81%. None of the 80 peptides screened showed a significant increase in viability compared to the negative controls.

**Figure 3:**
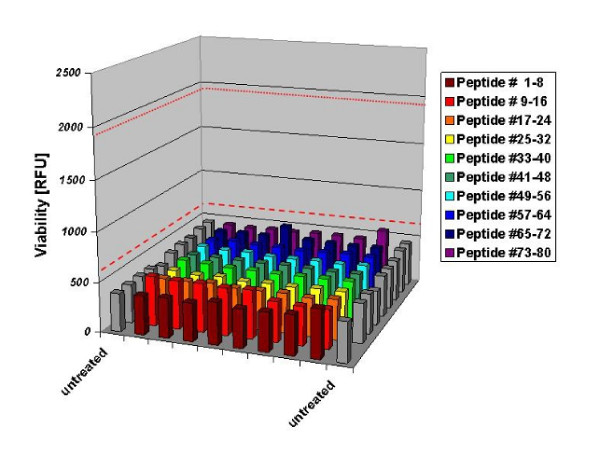
**Results of the G-CSF peptide screening at 10 μM**. Controls on each 96-well plate included two negative controls without G-CSF and two positive controls with a concentration of 0.1 nM and 3.4 pM G-CSF. Second positive control had to be adapted due to an increased DMSO concentration. The mean of the two positive controls over all plates are indicated as red lines (red dotted line = first positive control, red dashed line = second positive control). None of the 80 peptides showed a significantly increased viability compared to the negative controls. (n = 8 replicates each).

We conducted a second screen at a concentration of 10 μM per peptide. This resulted in an increase of the DMSO concentration to 2%, the highest level tolerated by the NFS-60 cell line as established before (data not shown). While at a 2% DMSO concentration the upper positive control remained at 0.1 nM as above, the lower positive control had to be elevated to 3.4 pM to show clear increase in viability. These concentrations were chosen as lower and upper positive control in the screen. Results are shown in Figure 3. Because of the tenfold increased DMSO content the average viability of the negative controls was clearly decreased relative to the previous screen. Positive controls again showed a significant increase in viability in all plates screened (mean values as red lines). None of the 80 peptides showed any significant increase in viability.

### Screening of peptides in a multiplex approach

As both screening approaches of single peptides with molarities of 1 μM and 10 μM were unsuccessful, we finally tried a multiplex approach, based on possibly necessary cooperative effects of different protein epitopes in order to activate the G-CSF receptor. As the cell line NFS-60 showed high sensitivity towards higher DMSO concentrations the maximum number of peptides to be tested per well were 20 at a total molarity of 10 μM (single concentration 0.5 μM). This resulted in a DMSO content of 2%.

**Figure 4:**
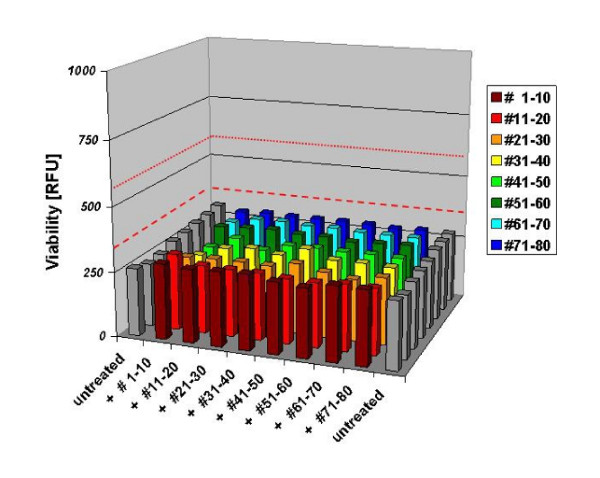
**Results of the multiplex peptide screening approach**. NFS-60 were incubated with four controls and eight peptide blocks. Controls included two negative controls without G-CSF and two positive controls with a molarity of 0.1 nM and 3.4 pM G-CSF (adapted control due to increased DMSO concentration). One peptide block consisted of ten peptides in increasing order and was combined with the remaining seven peptide blocks. None of the combinations showed an increase in viability at a total molarity of 10 μM compared to the negative controls. (n = 8 replicates each).

Figure 4 shows the results of this multiplex screening approach. Negative controls are represented as grey bars, whereas the positive controls are displayed in red lines. Negative controls displayed low viability comparable to the 10 μM single peptide screen. Although every possible combination of any two peptides derived from the G-CSF sequence was tested, the screen did also not yield any significant hits. Some of the peptide combinations appeared to have a small increase in viability but these elevations were never significant and never in the range of the lower positive control.

## Discussion

In the present study we have utilized the G-CSF dependence of the cell line NFS-60 to screen for agonistic peptides derived from the G-CSF human sequence. Although the screening system was highly sensitive and would have allowed the detection of an agonistic peptide at least 3.4 × 10 ^7^-fold less potent than the parent protein, we could not detect any positive hits. Also in a multiplex approach using a permutation of any combination of 10 different peptides we could not detect a positive block. A post-hoc power analysis of our screen revealed that it should have been possible to detect an increase in viability down to 10% with a power of 80%.

Reasons for this failure may either be technical problems, or principal in nature. We believe that the latter is true. The coverage of the G-CSF sequence was certainly dense with a frame shifted by 2 amino acids over the entire sequence. The purity of the synthesized peptides was reasonable with a minimum content of pure and intact peptide of 30%, and the peptides were all fully dissolved at the assayed concentrations.

It is possible that the length of the peptides was not sufficient. However, other agonistic peptides described for the EPO (Erythropoietin) receptor or NCAM (neural cell adhesion molecule) have 20 or less amino acids. Screening much longer peptides would also make less sense in view of their pharmaceutical advantage over the full-length protein.

An interesting point is the potential need for dimerising peptides. G-CSF itself dimerizes when binding to its receptor, and induces receptor dimerization [4-6]. Indeed, an engineered G-CSF dimer (F-627) appears more potent in vivo than the monomer form [33]. It has also been speculated that the nonpeptidyl small molecule SB 247464 acts because of its twofold rotational symmetry as a dimerization inducer of the G-CSF receptor [30]. It may therefore be advantageous in future approaches to utilize dimerizing peptides for such a screen.

## List of abbrevations

(G-CSF): Granulocyte-colony stimulating factor; (G-CSF-R): Granulocyte-colony stimulating factor receptor; (JAK): Janus kinase; (ALS): Amyotrophic lateral sclerosis; (BBB): blood-brain-barrier; (NCAM): neural cell adhesion molecule; (FGF): fibroblast growth factor, (EPO-R): Erythropoietin receptor; (M-CSF): Macrophage colony-stimulating factor; (IL-3): Interleukin-3.

## Competing interests

The authors are employees of SYGNIS Bioscience. This does not interfere with any aspects of this study.

## Authors' contributions

NC established, designed, and carried out the screening assay and analysis, and wrote the manuscript. AS conceived the study, contributed to analysis of the data, and wrote the manuscript. Both authors read and approved the final manuscript.

## Supplementary Material

Additional file 1 **Schematic design of the screening plates**. Description: 96-well plates were filled in a diagonal way to avoid evaporation effects and to get equal conditions for every sample. (A and B) Exemplary design of plates for 1 μM and 10 μM screening. Plates contained two negative controls, two positive controls and eight peptides with arising numbering. Second positive control had to be adapted due to increased DMSO content in the 10 μM screening. (C) Exemplary graphic of plates for the multiplex screening. Layout of controls was analogue to the 10 μM screening. Click here for file
