# Supplementary figures and images for: A screen for peptide agonists of the G-CSF receptor
Source: BMC Res Notes. 2011 Jun 15;4:194. doi: 10.1186/1756-0500-4-194 (PMC3132715; doi:10.1186/1756-0500-4-194)

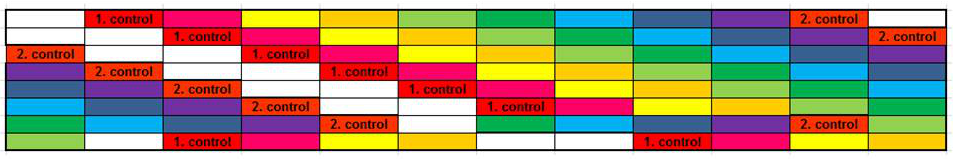

Supplement: Additional file 1 — Schematic design of the screening plates. Description: 96-well plates were filled in a diagonal way to avoid evaporation effects and to get equal conditions for every sample. (A and B) Exemplary design of plates for 1 μM and 10 μM screening. Plates contained two negative controls, two positive controls and eight peptides with arising numbering. Second positive control had to be adapted due to increased DMSO content in the 10 μM screening. (C) Exemplary graphic of plates for the multiplex screening. Layout of controls was analogue to the 10 μM screening. [file 1756-0500-4-194-S1.TIFF]
